# Differential maturation of the brain networks required for the sensory, emotional, and cognitive aspects of pain in human newborns

**DOI:** 10.1097/j.pain.0000000000003619

**Published:** 2025-06-18

**Authors:** Laura Jones, Dafnis Batalle, Judith Meek, A. David Edwards, Maria Fitzgerald, Tomoki Arichi, Lorenzo Fabrizi

**Affiliations:** a Department of Neuroscience, Physiology, and Pharmacology, University College London, London, United Kingdom; b Department of Forensic and Neurodevelopmental Science, Institute of Psychiatry, Psychology and Neuroscience, King's College London, London, United Kingdom; c Centre for the Developing Brain, School of Biomedical Engineering and Imaging Science, Kings College London, London, United Kingdom; d Elizabeth Garrett Anderson Obstetric Wing, University College London Hospitals, London, United Kingdom

**Keywords:** Neonatal pain, Pain imaging, Developing human connectome project, Cortical pain networks, Preterms, Pain connectome

## Abstract

Pain is multidimensional, including sensory-discriminative, affective-motivational, and cognitive-evaluative components. Although the concept of pain is learned through life, it is not known when and how the brain networks that are required to encode these different dimensions of pain develop. Using the 2 largest available databases of brain magnetic resonance images—the developing Human Connectome Project and the Human Connectome Project—we have mapped the development of the pain connectome—the neural network required for pain perception—in infants from 26 to 42 weeks of postmenstrual age (PMA, n = 372), compared with adults (n = 98). Partial correlation analysis of resting BOLD signal between pairwise combinations of 12 pain-related brain regions showed that overall functional connectivity is significantly weaker before 32 weeks PMA compared with adults. However, over the following weeks, significantly different developmental trajectories emerge across pain connectome subnetworks. The first subnetwork to reach adult levels in strength and proportion of connections is the sensory-discriminative subnetwork (34-36 weeks PMA), followed by the affective-motivational subnetwork (36-38 weeks PMA), while the cognitive-evaluative subnetwork has still not reached adult levels at term. This study reveals a previously unknown pattern of early development of the infrastructure necessary to encode different components of pain experience. Newborn neural pathways required for mature pain processing in the brain are incomplete in newborns compared with adults, particularly regarding the emotional and evaluative aspects of pain. The rapid age-related changes suggest that pain processing, and consequently pain experience, changes rapidly over this developmental period and unlikely to be the same as in adults, even at term.

## Abstract 2

Supplemental Digital Content is Available in the Text.

Neural pathways required to encode the sensory aspects of pain in the human infant brain mature in advance of those for emotional and evaluative aspects.

## 1. Introduction

Pain is a multidimensional experience and results from the interplay between contextual, intrinsic, and sensory factors. Pain perception is underpinned by the activation of a widespread network of brain regions, which together are responsible for the encoding of its sensory-discriminative, affective-motivational and cognitive-evaluative processes (see Table 1, supplemental digital content, http://links.lww.com/PAIN/C270). The sensory-discriminative component is primarily responsible for identifying and localizing the intensity and quality of pain, resulting from the ascending projections from the brain stem to the thalamus, and then to cortical regions such as the primary and secondary somatosensory cortices (SI and SII), which process sensory details, and the posterior insula, which contributes to sensory aspects of pain perception.^6,14,20,26,27,96,111^ The affective-motivational component is associated with the unpleasant and threatening nature of the stimulus resulting in the survival and emotional responses to pain. This involves the anterior cingulate cortex, anterior insula, and amygdala, with input from the thalamus.^1,18,20,22,61,71,79,109,111^ Finally, the cognitive-evaluative component reflects the appraisal, interpretation, and modulation of pain, engaging the prefrontal cortex (further subdivided into ventrolateral; dorsolateral; and orbitofrontal), anterior insula and midcingulate cortex, which contribute to attention, expectation, and executive control.^11,49,60,86,92,94,103,105,110^ The basal ganglia have been implicated in all subnetworks, and potentially function to integrate the different responses to pain.^16^

To function in an orchestrated fashion, these areas form preferential structural and functional connections with each other creating a network known as the *pain connectome*.^66^ For example, within the pain connectome, the anterior insula is predominantly connected to the ventrolateral prefrontal cortex and orbitofrontal cortex, a system associated with cognitive-affective aspects of pain, whereas the posterior insula is mainly connected with the primary and secondary somatosensory cortices, performing more sensory-discriminative functions.^108^ The activation of this network after a painful stimulus, such as laser or contact heat, results in characteristic electroencephalographic response whose components are correlated to stimulus intensity, saliency, and subjective pain report.^46,104,111^

In preterm neonates, noxious-evoked electroencephalographic responses to a clinically required heel lance change dramatically over the period equivalent to the third gestational trimester.^34^ Some components of this response are immature and only present at the beginning of this developmental period, some are transient and others only appear when approaching term equivalent age.^82,98^ The modulation of these components are related to various intrinsic and contextual factors such as sex, stress, behaviour, postnatal age, and parental holding.^19,57,58,102^ As the brain changes rapidly over the third gestational trimester (increase in gray matter volume and cortical gyrification, and maturation of thalamo-cortical and cortico-cortical functional/structural connectivity^28,39,56,63,91^), we hypothesised that these previously described changes in EEG nociceptive activity reflect the maturation of the underlying brain networks required for pain processing.

To test this hypothesis, we used the neonatal resting-state functional MRIs from the Developing Human Connectome Project (dHCP) database to characterise the developmental trajectory of functional connections within the pain connectome and compare these with the mature network configuration seen in adults from the Human Connectome Project (HCP).

## 2. Materials and methods

### 2.1. Neonatal and adult databases

Preprocessed fMRI data in native space^37^ from the open-access database of the Developing Human Connectome Project (dHCP third release)^50^ were used for this study. Data were acquired on a 3-T Philips Achieva scanner using a neonatal 32-channel receive coil and imaging system (RAPID Biomedical GmbH, Rimpar DE^50^). Subjects were scanned in natural sleep after feeding. High temporal resolution BOLD fMRI was acquired over 15 minutes and 3 seconds (2300 volumes) using a multislice gradient-echo planar imaging sequence (EPI) with multiband excitation optimized for neonatal scanning (multiband factor 9, repetition time 392 ms, 2.15 mm isotropic resolution).^78^ fMRI data were registered to subject-specific, high-resolution, motion-corrected^24^ T_2_-weighted structural images acquired during the same scan session (in-plane resolution 0.8 × 0.8 mm, slice thickness 1.6 mm overlapped by 0.8 mm, repetition time 12,000 ms, echo time 156 ms). Neonatal fMRI datasets were preprocessed using a dedicated neonatal pipeline.^37^ This includes correction of local distortion because of field inhomogeneity with *topup*^3^; intravolume and intervolume motion correction; and associated dynamic distortions correction using rigid-body realignment and slice-to-volume *eddy*.^4^ Twenty-four extended motion parameters together with independent components containing residual motion, multiband acquisition, and cardiorespiratory artefacts were regressed out using FSL FIX.^45^

Preprocessed adult fMRI data in standard MNI space were taken from the open-access WU-Minn Human Connectome Project database^99^ (HCP Young Adult S1200 data release, https://www.humanconnectome.org/study/hcp-young-adult/document/1200-subjects-data-release). Data were acquired on a 3-T scanner (customized Connectome Scanner adapted from a Siemens Skyra) using a standard 32-channel receive-only head coil.^100^ Subjects were scanned while relaxed with eyes open in a dark room. High temporal resolution BOLD fMRI was acquired over 15 minutes (1200 volumes) using a multislice gradient-echo EPI with multiband excitation (multiband factor 8, repetition time 720 ms, 2 mm isotropic resolution^36,72,85^). fMRI data were registered to subject-specific, high-resolution, motion-corrected T_1_-weighted structural images acquired during the same scan session (0.7 mm isotropic resolution, repetition time 2400 ms, echo time 2.14 ms^70^) and MNI template space. Adult fMRI datasets were preprocessed using a pipeline that includes spatial correction for distortions caused by gradient nonlinearity, motion correction through rigid body registration to the single-band reference image, correction for B0 distortion.^43^ Temporal processing involved high-pass temporal filtering and regression of motion and artifact time series using FSL FIX, incorporating 24 extended motion parameters. Data quality control was performed as part of automated preprocessing pipelines applied to the data before public release.^37,90^ The temporal signal-to-noise ratio of the neonatal and adult datasets is comparable and about 40 to 50.^37,90^ Framewise displacement was significantly different across the 7 age groups (1-way ANOVA, F[6, 463] = 8.79, *P* < 0.001); however, only the 38 to 40 and 40 to 42 weeks postmenstrual age (PMA) groups exhibited more motion compared with adults (Bonferroni-corrected pairwise comparisons, *P* < 0.001; *P* < 0.001) and to the 34 to 36 weeks PMA (*P* = 0.009; *P* = 0.003) and 36 to 38 weeks PMA (*P* = 0.040; *P* = 0.006) groups (see Table 2, supplemental digital content, http://links.lww.com/PAIN/C270).

### 2.2. Dataset inclusion

The dHCP database includes data from 807 infants (https://www.developingconnectome.org/). We excluded: (1) infants with postnatal age (PNA) >2 weeks, (2) infants with major brain abnormalities (radiology score of 4 or 5, which represent, eg, major lesions within the white matter), (3) data that did not pass the dHCP quality control assessment, as noted in the database documentation. The final sample consisted of 372 infants (26-42 weeks PMA, 0 to 14 days old, 43% female; Table 1).

**Table 1:** Infant subject demographics (n = 372).

Age group (N)	GA (wk)	PMA (wk)	PNA (d)	No. female	No. singleton births	Head circumference at birth (cm)	Birth weight (g)
All (372)	39 (24-41)	39 (26-42)	2 (0-14)	160 (43%)	317 (85%)	34 (21-38)	3170 (720-4800)
<32 (8)	28 (24-30)	29 (26-31)	12 (4-14)	2 (25%)	1 (13%)	27 (21-28)	1143 (720-1350)
32-34 (8)	32 (30-33)	33 (32-33)	5 (3-11)	5 (63%)	5 (63%)	29 (26-31)	1645 (1050-2280)
34-36 (34)	34 (32-35)	35 (34-35)	6 (1-14)	15 (44%)	15 (44%)	32 (29-34)	2120 (1250-3060)
36-38 (40)	36 (34-37)	37 (36-37)	4 (1-12)	19 (48%)	17 (43%)	32 (30-35)	2500 (1570-4100)
38-40 (100)	38 (36-39)	39 (38-39)	2 (0-13)	39 (39%)	91 (91%)	34 (30-37)	3175 (1820-4570)
40-42 (182)	40 (38-41)	41 (20-42)	2 (0-14)	80 (44%)	182 (100%)	35 (30-38)	3465 (2155-4800)

Values represent median (range) or total (%) for the number of females and singleton births.

GA and PMA values reflect completed weeks (wk, eg, 37.4 wk = 37 completed weeks).

GA, gestational age; PMA, postmenstrual age; PNA, postnatal age.

The 1200 Subjects Release (S1200) of the Human Connectome Project includes data from 1206 healthy young adult (https://www.humanconnectome.org/study/hcp-young-adult/document/1200-subjects-data-release). One hundred datasets were arbitrarily sampled for our study. Data were accepted if the participant had a resting-state fMRI of 15 minutes (1200 volumes) and had no issues identified during the HCP quality control process (eg, anatomical anomalies, structural segmentation errors) as specified in the database documentation and described in the reference manual of the data release. Two datasets were excluded because of shorter acquisition times. The final sample consisted of 98 adults (22-35 years old, 55% female). The proportion of female participants in each age group was not significantly different across newborns and adults (χ^2^ = 7.16, *P* = 0.306).

### 2.3. Pain connectome atlas

**Figure 1.**
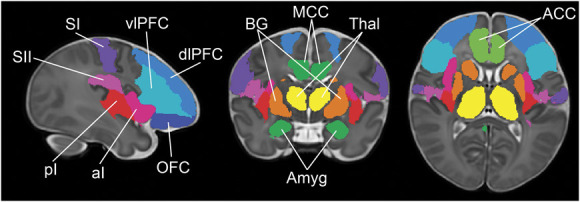
Pain connectome atlas. Masks of pain-related ROIs shown in sagittal, coronal, and axial planes, overlaid on the dHCP 40-week PMA T2 standard. Basal ganglia (BG, orange), thalamus (Thal, yellow), anterior and mid cingulate cortices (ACC, MCC, green), amygdala (Amyg, green), ventrolateral prefrontal cortex (vlPFC, cyan), dorsolateral prefrontal cortex (dlPFC, blue), orbitofrontal cortex (OFC, navy), primary and secondary somatosensory cortices (SI, purple; SII, magenta), and anterior and posterior insula (aI, magenta and pI, red).

The cortical regions involved in the pain connectome were identified as those areas consistently engaged in pain processing across adult studies.^6,11,27,51,73,104,112^ These include in both hemispheres: the thalamus, primary and secondary somatosensory cortices (SI and SII), anterior and posterior insula (aI and pI), anterior and mid-cingulate cortices (ACC and MCC), amygdala, basal ganglia (BG), orbitofrontal cortex (OFC), and ventrolateral and dorsolateral prefrontal cortices (vlPFC and dlPFC). Masks for these 12 pain-related regions of interest (ROIs) were based on an initial parcellation from a neonatal version^88^ of the automated anatomical labelling atlas (AAL),^97^ which has been adapted to the dHCP 40-week PMA T2 standard.^83,95^ Some ROIs were derived directly from the neonatal AAL without modification (thalamus, ACC, MCC, amygdala, BG [consisting of pallidum, putamen, and caudate], OFC [consisting of frontal superior, frontal mid and frontal inferior orbital cortices], vlPFC [consisting of frontal inferior operculum and frontal inferior triangularis], and dlPFC [consisting of frontal mid and frontal superior cortices]). SI and SII are not labelled in the AAL and had to be manually drawn. SI included the postcentral gyrus and the portion of the paracentral lobule posterior to the central sulcus. SII was the region of the rolandic operculum above the sylvian fissure. Finally, the insula was separated in anterior and posterior to the central sulcus.^2^ Other brain areas, such as the cerebellum, hippocampus, brainstem, and motor regions, are reportedly activated after noxious stimulation,^6,11,21,23,27,38,40,68,87^ but either not consistently or not in relation to the core pain processing aspects explored in this study (ie, sensory-discriminative, affective-motivational, and cognitive-evaluative) and therefore were not included in our analysis. The resulting pain connectome atlas, defined on the dHCP 40-week PMA T2 standard (Fig. 1), was resampled to infant native fMRI space by combining (1) the 40-week PMA to each PMA week standard (publicly available, https://git.fmrib.ox.ac.uk/seanf/dhcp-resources/-/blob/master/docs/dhcp-augmented-volumetric-atlas-extended.md) and (2) the standard to native fMRI warps (nonlinear registration based on a diffeomorphic symmetric image normalisation method [SyN]^8^ using ANTs, see França et al.^39^ for details). The same atlas was also resampled to the adult MNI 152 standard space using dHCP warps (publicly available, https://git.fmrib.ox.ac.uk/seanf/dhcp-resources/-/blob/master/docs/dhcp-augmented-volumetric-atlas-extended.md). Atlas resampling was visually checked for accuracy for each individual.

### 2.4. Tissue segmentation

To select the gray matter portion of the ROIs for functional connectivity analysis, a neonatal tissue segmentation template (dHCP Augmented Volumetric Atlas^83^) was resampled to infant fMRI native space using the same warps of the AAL resampling. For adults, an open-access MNI tissue segmentation template (https://github.com/Jfortin1/MNITemplate) was used.

### 2.5. Functional connectivity analysis

We first calculated the BOLD signal over a 15-minute segment averaged across voxels for each pain-related ROI (gray matter only) in each subject. We then calculated the Pearson partial correlation coefficients (*r*) between each possible pair of ROIs (n = 66 connections) and took the absolute *r* value. This is a measure of the linear relationship between the BOLD signals of the 2 ROIs while accounting for the effect of all the others. The *r* values can be positive or negative depending on the phase difference between the BOLD time series from the 2 ROIs which might represent distinct physiological processes.^44^ However, the strength of functional connectivity, independently of its nature, can be measured as the absolute value of the correlation coefficients, which also has good reproducibility.^80^ Absolute *r* values from homologous ROI pairs in the 2 hemispheres were then averaged.

Spurious connectivity values were removed using conventional z-thresholding for each connection. In the infant group, to account for the increase in connectivity during the equivalent of the third gestational trimester, we applied Cook distance. For each connection *C*, we calculated the distance between connectivity value for each infant *i* (*r*_*Ci*_) and the predicted values from the linear relationship between (*r*_*Ci*_) and PMA. *r* values with a Cook distance exceeding 3× the average Cook distance were discarded. For adults, *r* values that exceeded 3× the SD from the average *r* value were discarded; 6.4% and 0.5% of infant and adult connectivity values were discarded (see Figure 1, supplemental digital content, http://links.lww.com/PAIN/C270).

We then normalised each connectivity value (*r*_*Ci*_) for each infant *i* and connection *C* by the average adult ${\hat{r}}_{CA}$ value for that connection to obtain *r-norm*_*Ci*_:

(1) $${r‐norm}_{Ci}=\frac{r_{Ci}}{{\hat{r}}_{CA}}$$

This can be interpreted as degree of adult-like functional connectivity. To determine the presence/absence of a connection, we compared all *r-norm*_*Ci*_ to the average *r-norm* of the thalamus-SI connection in the youngest infant group (<32 weeks PMA), which is known to be already functional at this age.^7,107^ *r-norm*_*Ci*_ below this reference value were set to 0 (ie, absent connection).

### 2.6. Statistical analysis

**Figure 2.**
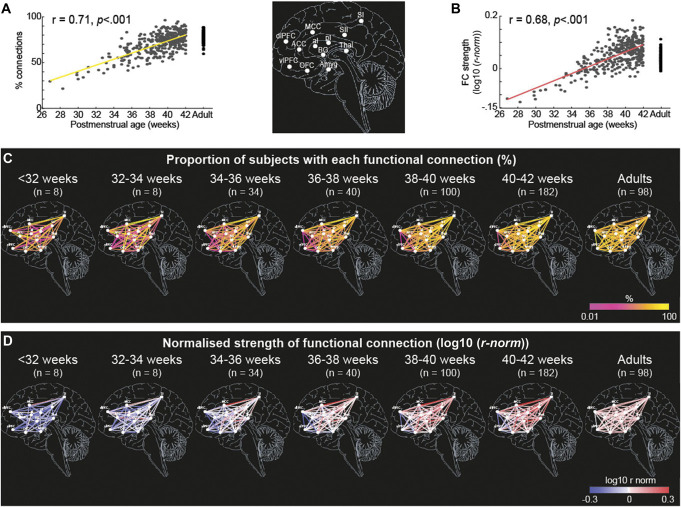
Development of functional connectivity across the pain connectome over of the equivalent of the third gestational trimester. Proportion of functional connections within the pain connectome (A and C) and their strength (B and D) over the equivalent of the third gestational trimester and adults. Data presented as: (A) scatter plot and linear regression (solid lines) of proportion of connections within the pain connectome and (B) average strength for each subject; (C) maps of proportion of subjects with each connection and (D) average strength of those connections for each ROI (white dots) pairs in 6 age groups (<32 ( n = 8), 32-34 (n = 8), 34-36 (n = 34), 36-38 (n = 40), 38-40 (n = 100), 40-42 (n = 182) weeks PMA) and in adults (n = 98).

To assess the development of adult-like functional connectivity within the pain connectome over the equivalent of the final gestational trimester, we assessed the relationship (linear regression) between PMA and (1) proportion of present functional connections (% *r-norm* values above 0) of all possible pain-related connections for each subject, and (2) the average strength of functional connectivity, log_10_(*r*-*norm*), of all pain-related connections for each subject. We then compared the average proportion of functional connections and strength of functional connectivity at different PMAs (<32, 32-34, 34-36, 36-38, 38-40, 40-42 weeks PMA; see Figure 2, supplemental digital content, http://links.lww.com/PAIN/C270) with those in adults to determine if and when functional connectivity reached adult-like values (Dunnet corrected *t* tests). To then compare the developmental trajectory within the sensory (SI, SII, thalamus, BG, and posterior insula), affective (anterior insula, ACC, thalamus, amygdala, and BG), and cognitive (dlPFC, vlPFC, OFC, MCC, BG, and anterior insula) subnetworks, we performed 2-way ANOVA (age × sub-network) for (1) the proportion of functional connections and (2) strength of functional connectivity, followed by Tukey corrected pairwise comparisons. Finally, to assess the degree of adult-like functional connectivity of each subnetwork by late-term age (40-42 weeks PMA), we compared the average strength of functional connectivity of each connection within each subnetwork between neonates and adults (FDR corrected *t* tests).

## 3. Results

To assess the developmental trajectory of the pain connectome over the equivalent of the third gestational trimester, we measured changes in functional connectivity between 12 pain-related regions of interest (ROI, Fig. 1) in term- and preterm-born infants scanned between 26 and 42 weeks postmenstrual age (PMA) from the developing Human Connectome Project (dHCP, ^50^ N = 372), and compared them with those in adults from the Human Connectome Project, (HCP,^99^ N = 98). To ensure that the results reflected intrinsic maturation of cortical networks and were not affected by ex utero experience, only data from infants <2 weeks old (<2 weeks postnatal age, PNA) were used.

### 3.1. Functional connectivity within the pain connectome increases over the equivalent of the final gestational trimester

We first examined the overall changes in functional connectivity across the whole pain connectome between 26 and 42 weeks PMA.

The data show that there is a significant increase in the percentage of functional connections present (*R*^2^ = 0.50, *P* < 0.001, Fig. 2A) across the pain connectome and a significant increase in the strength of those connections with postmenstrual age (*R*^2^ = 0.46, *P* < 0.001, Fig. 2B). Thus, 50% of the variance in the percentage of functional connections present and 46% of the variance in their strength is explained by age. No connections were present in neonates that were not present in adults.

To determine the postmenstrual age at which the proportion of subjects with each connection (referred to as *proportion of connections* from now on, Fig. 2C) and strength of connections (normalised to adult average values, referred to as *strength of connections* from now on, Fig. 2D) reach adult levels, we compared average values across the pain connectome within 6 age groups (<32, 32-34, 34-36, 36-38, 38-40, and 40-42 weeks PMA) with average value in adults. The average proportion of connections was significantly lower in all, but the oldest neonates compared with adults (Dunnett corrected *t* tests, *P* < 0.01; see Figure 2a, supplemental digital content, http://links.lww.com/PAIN/C270). Furthermore, the average strength of connections differed significantly between each of the 6 neonatal age groups and adults (Dunnett corrected *t* tests, *P* < 0.01). Although the overall strength of connections was significantly lower than that in adults up to 38 weeks PMA, the strength of connections between 38 and 42 weeks PMA significantly exceeded adult levels (see Figure 2b, supplemental digital content, http://links.lww.com/PAIN/C270).

### 3.2. The development of connectivity is nonuniform across different functional subnetworks of the pain connectome

Inspection of Figure 2 indicated that the developmental profile of different functional connections was not uniform across the pain connectome (Fig. 2C and D, see Table 3, supplemental digital content, http://links.lww.com/PAIN/C270). Some connections reached adult-like proportion and strength at an early PMA, whereas other connections remained weaker even at term age.

To explore the uneven development of the connections within the pain connectome, we divided the overall network into the 3 subnetworks responsible for sensory-discriminative, affective-motivational, and cognitive-evaluative processing of a noxious stimulus, according to adult studies (see Methods for details). We then assessed the development of each subnetwork (in proportion and strength of connections) across 7 age groups (<32, 32-34, 34-36, 36-38, 38-40, and 40-42 weeks PMA and adult).

The proportion and strength of connections was overall significantly different across subnetworks and age groups (2-way ANOVA, main effect of subnetwork on proportion: F[2, 1389] = 47.4, *P* < 0.001 and strength: F[2, 1387] = 27.01, *P* < 0.001; main effect of age group on proportion: F[6, 1389] = 110, *P* < 0.001 and strength: F[6, 1387] = 77.91, *P* < 0.001) and, most importantly, there was a significant interaction between subnetworks and age groups (proportion: F[1, 21389] = 5.27, *P* < 0.001, variance = 2.86%; strength: F[1, 21387] = 3.50, *P* < 0.001, variance = 2.15%), confirming that the developmental trajectory of functional connectivity was not homogeneous across subnetworks.

**Figure 3.**
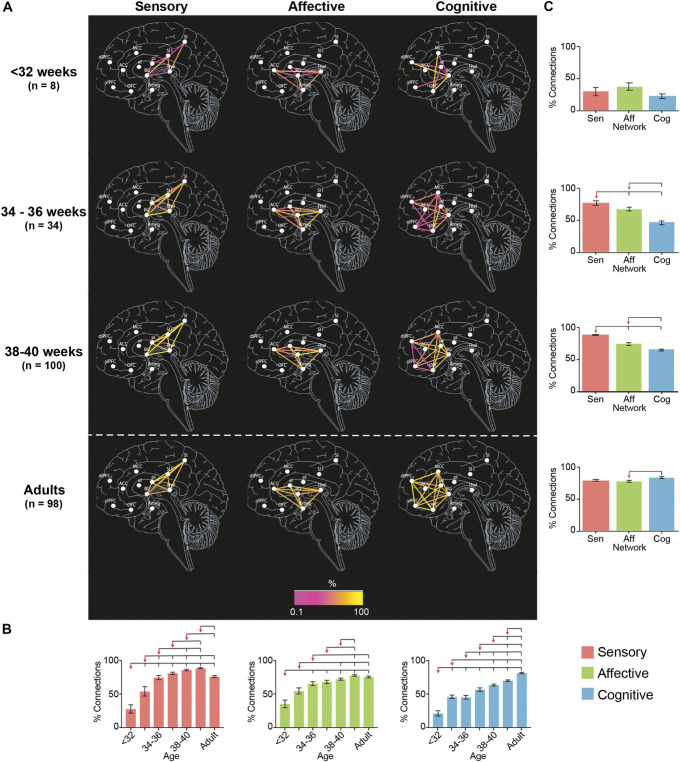
Proportion of connections within the sensory, affective, and cognitive subnetworks of the pain connectome. Maps of the proportion of connections within the sensory, affective, and cognitive subnetworks at <32, 34-36, and 38-40 weeks PMA and adults (A). Effect of PMA within each subnetwork (B) and of subnetwork at each age group (C) on the proportion of connections. Overlying brackets denote significant pairwise differences between the group marked by the red arrow and the others. Error bars represent SEM. Full inferential statistics in see Table 4 and 5, supplemental digital content, http://links.lww.com/PAIN/C270.

**Figure 4.**
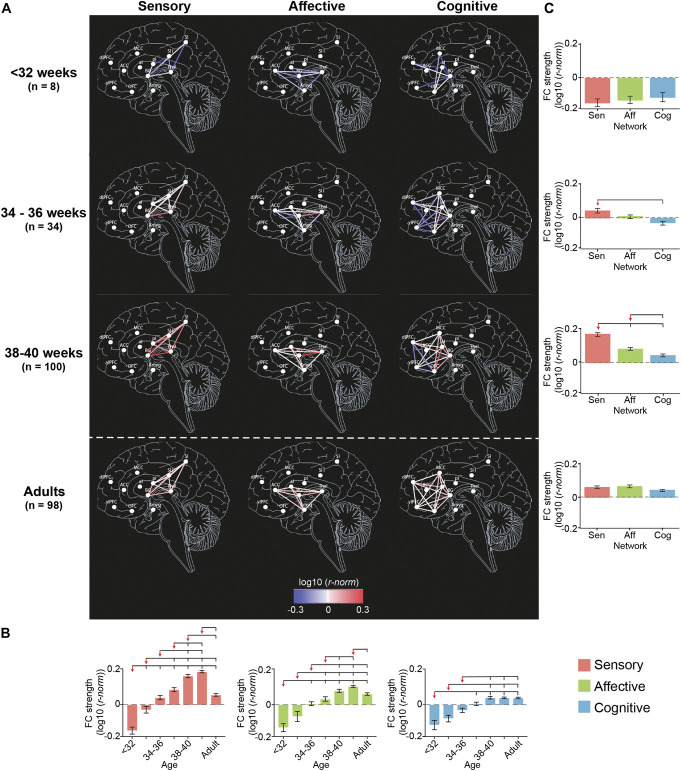
Strength of connections within the pain connectome subnetworks. Maps of the strength of connections (*r*-*norm*) within the sensory, affective, and cognitive subnetworks at <32, 34-36, and 38-40 weeks PMA and adults (A). The color scale represents the average strength of each functional connection across subjects within each group in log scale. Effect of PMA within each subnetwork (B) and of subnetwork at each age group (C) on the strength of connections. Overlying brackets denote significant pairwise differences between the group marked by the red arrow and the others. Error bars represent SEM. Full inferential statistics are in Table 4 and 5, supplemental digital content, http://links.lww.com/PAIN/C270.

Before 34 weeks PMA, all subnetworks were in similar conditions (Figs. 3 and 4, see Table 4 and 5, supplemental digital content, http://links.lww.com/PAIN/C270). The proportion and the strength of connections was not significantly different across subnetworks (Tukey corrected pairwise comparison, *P* > 0.097) and was significantly lower than in adults for all subnetworks (Tukey corrected pairwise comparison, *P* < 0.001). However, there was already a significant increase in proportion for the sensory and cognitive subnetworks and in strength of connections for the sensory subnetwork between <32 and 34 weeks PMA.

This steady, but inhomogeneous, increase in proportion and strength of connections continued after 34 weeks PMA (Figs. 3 and 4, see Table 4 and 5, supplemental digital content, http://links.lww.com/PAIN/C270).

The sensory subnetwork: (1) had significant increases in proportion and strength of connections with age; (2) overtook the other 2 subnetworks in proportion of connections from 34 weeks PMA and in strength of connections from 36 weeks PMA; (3) reached adult levels in proportion and strength of connections at 34 to 36 weeks PMA and (4) exceeded adult levels between 38 to 42 weeks PMA.

The affective subnetwork (1) had significant increases in proportion and strength of connections with age; (2) overtook the cognitive subnetwork in proportion of connections from 34 weeks PMA and in strength of connections from 38 weeks PMA, but was always lower than the sensory subnetwork; (3) reached adult levels in proportion and strength of connections at 36 to 38 weeks PMA; and (4) never exceeded adult level of proportion of connections but exceeded adult level of strength of connections at 40 to 42 weeks PMA, albeit to a lesser degree than for the sensory subnetwork (sensory: mean difference [95% CI] between 40 to 42 weeks PMA and adult = 0.11 [0.09-0.13]; affective: 0.04 [0.01-0.06]; see Table 4, supplemental digital content, http://links.lww.com/PAIN/C270). Within the affective subnetwork, the slowest connections to develop were those between the anterior insula and the anterior cingulate cortex and the amygdala, which were not present in any subject before 32 weeks PMA (see Table 3, supplemental digital content, http://links.lww.com/PAIN/C270).

The cognitive subnetwork lagged behind the other 2 subnetworks: (1) had significant increases in proportion and strength of connections with age, however (2) never overtook the other 2 subnetworks; (3) never reached adult levels of proportion of connections but reached adult level of strength of connection at 36 to 38 weeks PMA; and (4) never exceeded adult levels in proportion or strength of connections. Within the cognitive subnetwork, the slowest connections to develop were those between the ventrolateral and dorsolateral prefrontal cortex, which were not present in any subject before 36 weeks PMA (see Table 3, supplemental digital content, http://links.lww.com/PAIN/C270).

### 3.3. Nonuniform maturity of functional connections within pain connectome subnetworks at the end of the gestational period

To assess the level of maturity of each connection by the end of the gestational period, we compared the average strength of connection at 40 to 42 weeks PMA with that in adults (FDR corrected Student *t* test, *P* < 0.05). Within the sensory subnetwork, 70% of connections were significantly stronger in late-term infants compared with adults, and no connections were stronger in adults (see Figure 3, supplemental digital content, http://links.lww.com/PAIN/C270). Within the affective subnetwork, 40% of connections were significantly stronger and 10% weaker and within the cognitive subnetwork, 20% of connections were significantly stronger, whereas 40% were weaker in late-term infants compared with adults. Notably, the weaker connections were within prefrontal ROIs or connecting to these areas.

## 4. Discussion

In adults, pain perception is related to the activation of a widespread network of brain regions,^6,11,27,96^ underpinned by a grid of local and cross-cortical connections, known as the pain connectome.^66^ Functional connections within this network are altered in various pain conditions^9,55,69,75^ and their strength relates to pain perception,^9,75^ suggesting that, for normal pain processing, the pain connectome must be functionally intact. Here we show that this infrastructure for pain is significantly weaker at the beginning of the third gestational trimester compared with adults, follows an uneven developmental trajectory and does not reach mature configuration even at term age. Neonatal and adult fMRI data at rest were compared to assess the availability of pain connectome subnetworks involved in the sensory-discriminative, affect-motivational, and cognitive-evaluative aspects of pain processing at different developmental stages. Until 34 weeks postmenstrual age (PMA), all pain connectome subnetworks exhibited significantly lower proportions and strengths of functional connections compared with adults, with distinct developmental trajectories afterward. The sensory subnetwork developed faster, reaching adult levels at 34 to 36 weeks PMA and ultimately showing higher proportion and average strength of connections than adults at term age (70% of connections significantly stronger than adult), whereas the affective subnetwork reached adult levels later (36-38 weeks PMA) and on average exceeded adult strength levels at term age (40% of connections significantly stronger and 10% weaker than adult). The cognitive subnetwork lagged behind the other 2 networks, on average reaching adult strength levels at 36 to 38 weeks PMA but failing to reach adult proportion of connections even by term age (20% of connections significantly stronger and 40% weaker than adult). The fact that at no point over the equivalent of the third trimester of gestation does the pain connectome assume an adult-like configuration suggests that pain processing cannot completely engage those necessary connections and is therefore unlikely to be the same as in adults, even at term. The rapid age-related changes suggest that pain processing, and consequently pain experience, changes rapidly over this developmental period.

The inhomogeneous maturation of the pain connectome is reflected in changes in noxious-evoked cortical activity over the equivalent of the last trimester of gestation. Cortical responses to a skin-breaking stimulus in a preterm infant are present from 28 weeks PMA, but some components are transient and others only appear when approaching term-equivalent age.^34,81,98^ The modulation of these components is also related to different intrinsic and contextual factors,^19,57,58,102^ suggesting that they represent the activation of distinct cortical processes that are likely to mature at different times.

The increase in proportion and strength of functional connections is likely driven by axonal growth, dendritic arborization, synaptogenesis, and myelination occurring first in the subplate and then in the cortical plate.^31,47,63,64,68,101^ The developmental exuberance of some of these processes—such as the growth of transient axonal projections and the formation of temporary axonal and dendritic branches, synapses, and dendritic spines—leads to an initial excess of connections.^53^ These are subsequently pruned based on position molecular cues and activity explaining the functional hyperconnectivity observed in some connections compared with adults.^52^ Our results are consistent with the regional heterogeneity of these changes: disappearance of the subplate,^63–65^ synaptogenesis, and myelination^10,30,112^ occur earlier in sensorimotor areas than in the associative fibre bundles that are developing with a medio-lateral and caudo-rostral gradient.^29,74^

Hyperconnectivity in the sensory network might imply poor localization of pain sources. Indeed, responses to a heel lance stimulus in the neonatal SI include somatotopic areas, which in adults represent the hand.^59^ Most connections within the affective subnetwork are functionally available by 32 weeks PMA, except for the anterior insula–amygdala and anterior insula–ACC. The amygdala is involved in emotional memory, and the autonomic and somatic responses to threatening stimuli,^61^ whereas ACC and anterior insula are implicated in bodily and emotional awareness.^20^ Together, activity within and structural connectivity between these regions are related to pain awareness and aversion.^18,79,108,110^ These functions might therefore be immature before adult-like connectivity in this subnetwork is reached at 36 to 38 weeks PMA. However, the full pain experience is dependent on sensory-affective integration. Communication from SI to ACC is implicated in this integration, and tighter coupling between these areas is related to stronger aversive responses and more accurate noxious vs innocuous sensory discrimination.^89,93^ Here, we found that this connection is not functional until 32 to 34 weeks PMA, reaching adult-like connectivity only by 36 to 38 weeks PMA. In the cognitive subnetwork, over 50% of functional connections are not present until 32 to 34 weeks PMA, and only 75% of connections are present afterward. Intra-PFC connections are the last to develop and connectivity here remains weak at term. The PFC modulates the impact of, and attaches meaning to, sensations and emotions^15,77,84^ and is considered necessary for conscious perception and self-report.^12^ However, unconscious sensory registration can still initiate autonomic and behavioral survival responses and may still have long-term consequences because of implicit memories of aversive stimuli.^42^ Thus, as the PFC is largely unconnected over the third gestational trimester, neonates may not have conscious awareness or cognitive control of a noxious stimulus but may still have implicit memories after sensory and limbic activation.

Our results demonstrate that the pain connectome does not have the same basic functional architecture as in adults even at term. Indeed, noxious-evoked activity still maintains substantial differences from that in adults at this age,^35^ and resting-state networks representing primary sensory functions appear mature at term while those involving higher-order association areas remain fragmented and lack the contribution of the frontal cortices.^33,41,62^ The pain connectome is therefore likely to undergo a phase of pruning and refinement after birth, resulting in its mature adult configuration.^54^ Functional maturation by fibre pruning and myelination occurs from 36 weeks PMA in sensorimotor pathways (sensory-discriminative), however only occurs from 5 weeks post-term age for limbic fibres (affective-motivational) and from 10 weeks post-term age for frontal association fibres (cognitive-evaluative).^13,30,31,112^ Although thalamo-cortical projection and some limbic fibres (cingulum) are fully developed by the end of the first year, the association fibres continue to develop for up to 2 decades.^29^

Our study investigates the emergence of functional connections within the pain connectome which might differ from that of its structure. There is a clear overlap between the neonatal functional and structural connectivity layout,^48^ and both connectomes develop hierarchically, progressing from sensory to associative and from local to long-range connections,^17^ suggesting that the structural dimension of the pain connectome could be developing in parallel to its functional connections. Nevertheless, in future studies, exploring the anatomical maturation of the pain connectome could provide further information about the heterogeneity of its development and potential period of vulnerability for specific connections.

In this study, we focused on the core aspects of pain processing—sensory-discriminative, affective-motivational, and cognitive-evaluative components—and clustered brain regions accordingly. These clusters were based on a thorough review of the literature and widely accepted categorizations; however, it is important to recognize that the boundaries between these components are not always distinct. Many regions, such as the anterior insula and prefrontal cortex, contribute to multiple aspects of pain processing and may be involved in more than one subnetwork. This overlapping functionality suggests that the categorization into subnetworks should be viewed as a simplified model. Further research may refine our understanding of the interactions between these brain regions, particularly in the context of developmental trajectories. To enhance reproducibility and facilitate interpretation of our findings within the broader connectivity literature, we used data from 2 of the largest open-source imaging cohorts available. Although acquisition parameters were consistent within neonatal age groups, differences in protocol and hardware between neonates and adults—optimized for their respective age groups—may have influenced infant-adult comparisons, despite comparable signal-to-noise ratios across datasets. Including control groups within each cohort in future analyses could help mitigate these potential confounding effects.

We have demonstrated that functional connectivity within the pain connectome increases over the equivalent of the third trimester of gestation but remains immature at term. The development is heterogenous across different subnetworks such that sensory and affective networks are strongly connected at term, whereas the prefrontal cortex within the cognitive network is weakly connected. These data suggest that preterm neonates can encode sensory features of a noxious stimulus but are less able to process aversion to that stimulus and are not capable of conscious appraisal. The rapid development of the sensory network is likely driven by the early development of spinal cord nociceptive circuits in preterm infants^5,25^ and may explain the vulnerability of sensory processing behaviour to untimely clinical procedures experienced by infants born very preterm.^32^ The later maturation of emotional networks may explain the developmental vulnerability of pain emotion processing to early life injury and stress.^106^ The protracted development of the cognitive network suggests an extended period of wider vulnerability beyond term.^76^ Our results add new insights into the cortical infrastructures available for pain processing in preterm infants and into our understanding of pain in this vulnerable population.

## Conflict of interest statement

The authors have no conflict of interest to declare.

## Supplemental digital content

Supplemental digital content associated with this article can be found online at http://links.lww.com/PAIN/C270.

## Supplementary Material

SUPPLEMENTARY MATERIAL
