# Supplementary material for: Differential maturation of the brain networks required for the sensory, emotional, and cognitive aspects of pain in human newborns
Source: Pain. 2025 Jun 18;166(10):e351–62. doi: 10.1097/j.pain.0000000000003619 (PMC7617847; doi:10.1097/j.pain.0000000000003619)
Supplement: SUPPLEMENTARY MATERIAL [file jop-166-e351-s001.pdf]

Supplementary Information

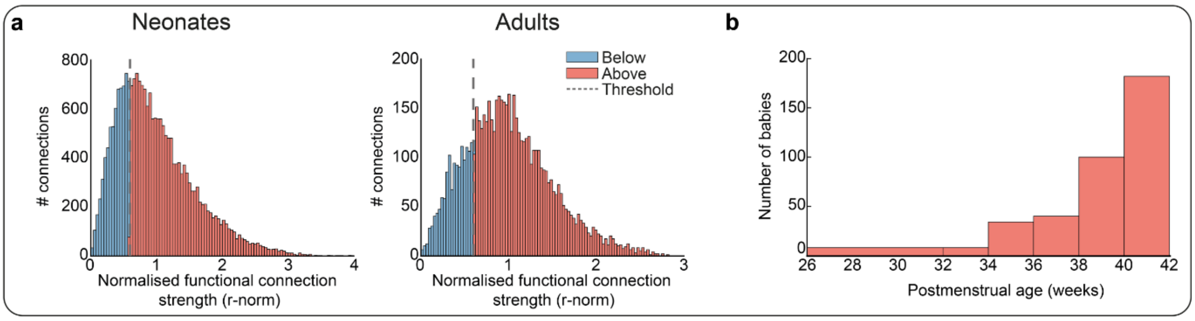

Supplementary Figure 1. Distributions of normalised (to adult) functional connection strength and infant postmenstrual age. Histogram of the normalised functional connection strength for all neonatal and adult connections (a). Grey dashed line denotes the reference value to determine the absence/presence of a connection (average functional connectivity of the thalamus to S1 connection in the youngest postmenstrual age (PMA) group (26-32 weeks)). Number of neonates in each PMA group (b): 26-32 (N = 8), 32-34 (N = 8), 34-36 (N = 34), 36-38 (N = 40), 38-40 (N = 100), 40-42 (N = 182).

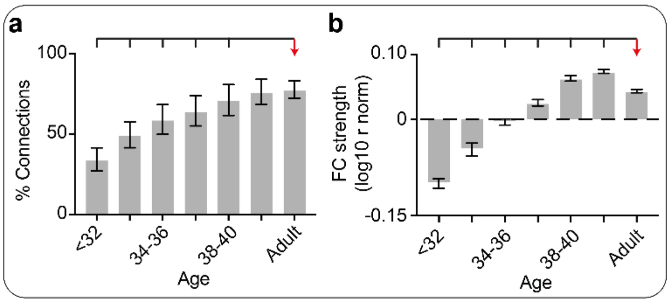

Supplementary Figure 2. Proportion and strength of functional connections within the pain connectome compared to adults. Average proportion of functional connections present across the pain connectome (a), and average strength of functional connectivity (b) across subjects for each of the 7 age groups (26-32 weeks PMA (N = 8), 32-34 (N = 8), 34-36 (N = 34), 36-38 (N = 40), 38-40 (N = 100), 40-42 (N = 182), Adult (N = 98)). Overlying brackets denote significant pairwise differences between adults and each neonatal age group. Error bars represent standard error of the mean.

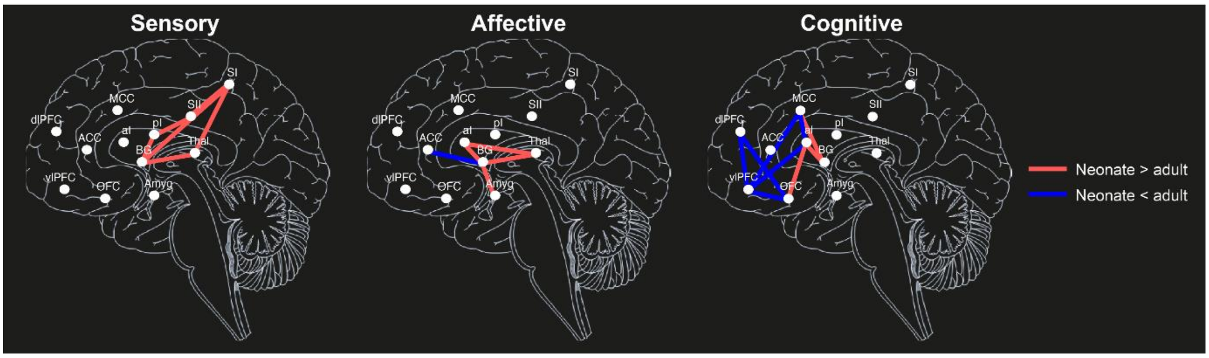

Supplementary Figure 3. Strength of connections within subnetworks in late-term neonates compared to adults. Comparison of the average strength of connectivity ($\log_{10}$ (r-norm)) for each connection within the 3 subnetworks (sensory, affective, and cognitive) between late-term neonates (40-42 weeks PMA) and adults. Connections with significantly different strength (FDR correct Student's t-tests) are denoted in blue (neonates have weaker connectivity) and red (neonates have stronger connectivity).

Supplementary Table 1. Summary the literature and reviews underpinning the selection of Region of Interests (ROIs) included in our analysis and subdivision of the pain connectome in the sensory-discriminative, affective-motivational and cognitive-evaluative subnetworks. The full references are in the reference list of the main paper. SI = Primary Somatosensory Cortex; SII = Secondary Somatosensory Cortex; PFC = Prefrontal Cortex (dl = dorso-lateral; vl = ventro-lateral); OFC = Orbitofrontal Cortex; ACC = Anterior Cingulate Cortex; MCC = Midcingulate Cortex.

ROI	Sub-regions	References for involvement in sub-networks	References for involvement in pain
Thalamus		*Sensory*<br>Craig et al., 2006<br>DaSilva et al., 2002<br><br>*Affective*<br>Aziz and Ahmad, 2006<br>Bushnell and Duncan, 1989<br>Mindaye et al., 2024	Apkarian et al., 2005<br>Aziz and Ahmad, 2006<br>Bushnell and Duncan, 1989<br>Bushnell et al., 2013<br>Coghill et al., 1999<br>Fomberstein et al., 2013<br>Jones et al., 1991<br>Mindaye et al., 2024<br>Tracey and Mantyn, 2007<br>Wager et al., 2013<br>Weich et al., 2014<br>Xu et al., 2020
SI		*Sensory*<br>Bingel et al., 2004<br>DaSilva et al., 2002<br>Peltz et al., 2011<br>Rainville et al., 1992	Apkarian et al., 2005<br>Bushnell et al., 2013<br>Coghill et al., 1999<br>Davis and Moayedi, 2013<br>Davis et al., 2013<br>Fomberstein et al., 2013<br>Iannetti and Mouraux, 2010<br>Iannetti et al., 2018<br>Kuyci et al., 2017<br>Mouraux and Iannetti, 2018<br>Ploner et al., 2017<br>Talbot et al., 1991<br>Tracey & Mantyn, 2007<br>Weich et al., 2014
SII		*Sensory*<br>Bingel et al., 2004<br>Maihofner et al. 2006<br>Vogel et al., 2003	Apkarian et al., 2005<br>Brooks et al., 2002<br>Bushnell et al., 2013<br>Coghill et al., 1999<br>Davis and Moayedi, 2013<br>Davis et al., 2013<br>Fomberstein et al., 2013<br>Iannetti and Mouraux, 2010<br>Iannetti et al., 2018<br>Kuyci et al., 2017<br>Mouraux and Iannetti, 2018

			Ploner et al., 2017<br>Talbot et al., 1991<br>Tracey & Mantyn, 2007<br>Wager et al., 2013<br>Weich et al., 2014<br>Xu et al., 2020
Insula	Anterior, medial, & posterior<br>Anterior/posterior delineation most common and is most suitable for neonates (Alcauter et al., 2015)	*Posterior → Sensory*<br>Albanese et al., 2007<br>Oertel et al., 2012<br>Raij et al., 2005<br>Wiech et al., 2014<br>*Anterior → Affective/Cognitive*<br>Kurth et al., 2010<br>Mitchell et al., 2010<br>Phillips et al., 2003<br>Ploner et al., 2011<br>Taniguchi et al., 2022<br>Tracey & Mantyn, 2007<br>Wiech et al., 2010<br>Wiech et al., 2014	Apkarian et al., 2005<br>Bastuji et al., 2016<br>Brooks et al., 2002<br>Bushnell et al., 2013<br>Coghill et al., 1999<br>Davis and Moayedi, 2013<br>Davis et al., 2013<br>Fomberstein et al., 2013<br>Iannetti and Mouraux, 2010<br>Iannetti et al., 2018<br>Kuyci et al., 2017<br>Mouraux and Iannetti, 2018<br>Ploner et al., 2017<br>Tracey & Mantyn, 2007<br>Wager et al., 2013<br>Weich et al., 2014<br>Xu et al., 2020
PFC	dlPFC, vlPFC cited most when PFC is subdivided	*Cognitive*<br>Lorenz et al., 2002 [lPFC]<br>Peltz et al., 2011<br>Salomons et al., 2007 [vlPFC]<br>Sridharan et al., 2008 [dlPFC]<br>Wiech et al., 2006 [vlPFC]	Apkarian et al., 2005<br>Bastuji et al., 2016 [dlPFC]<br>Bushnell et al., 2013<br>Coghill et al., 1999<br>Davis et al., 2013<br>Fomberstein et al., 2013<br>Ploner et al., 2017<br>Tracey & Mantyn, 2007<br>Wager et al., 2004 [dlPFC]<br>Wager et al., 2013<br>Weich et al., 2014 [dlPFC, vlPFC]<br>Xu et al., 2020 [lPFC]
OFC		*Cognitive*<br>Kahnt et al., 2010	Bastuji et al., 2016<br>Wager et al., 2004<br>Weich et al 2014
ACC		*Affective*<br>Phillips et al., 2003<br>Rainville et al., 1992<br>Weich et al., 2010	Apkarian et al., 2005<br>Bastuji et al., 2016<br>Brooks et al, 2002<br>Bushnell et al., 2013<br>Coghill et al., 1999<br>Davis and Moayedi, 2013<br>Davis et al., 2013

			Fomberstein et al., 2013
			Iannetti and Mouraux, 2010
			Iannetti et al., 2018
			Jones et al., 1991
			Kuyci et al., 2017
			Mouraux and Iannetti, 2018
			Ploner et al., 2017
			Talbot et al., 1991
			Tracey & Mantyn, 2007
			Wager et al., 2013
			Weich et al., 2014
			Bastuji et al., 2016
			Bushnell et al., 2013
			Davis and Moayedi, 2013
		Cognitive	Davis et al., 2013
MCC		Hu et al., 2019	Fomberstein et al., 2013
		Shackman et al., 2011	Jones et al., 1991
		Vogt et al., 2016	Kuyci et al., 2017
			Ploner et al., 2017
			Weich et al., 2014
			Xu et al., 2020
			Bastuji et al., 2016
		Affective	Bushnell et al., 2013
Amygdala		Gracely et al., 2004	Fomberstein et al., 2013
		Kang et al., 2021	Tracey & Mantyn, 2007
		Tracey & Mantyn, 2007	Weich et al., 2014
			Xu et al., 2020

Supplementary Table 2. Mean framewise displacement (and standard deviation, SD). As reported in the quality control databases of the developing Human Connectome Project (https://pubmed.ncbi.nlm.nih.gov/32866666/) and the 1200 Subjects Release (S1200) of the Human Connectome Project (https://github.com/dmascali/HCP_resting-state_QC/tree/master).

**Age group (N)**	**Framewise displacement Mean (SD)**
<32 (8)	0.31 (0.16)
32-34 (8)	0.19 (0.11)
34-36 (34)	0.18 (0.15)
36-38 (40)	0.19 (0.12)
38-40 (100)	0.32 (0.28)
40-42 (182)	0.28 (0.18)
Adults (98)	0.16 (0.06)

Supplementary Table 3. Average normalised connection strength within the pain connectome. Average normalised (to adult average values) connection strength (log_{10} (r-norm)) and standard error for every connection (basal ganglia [BG], Thalamus [Thal], anterior and mid cingulate cortices [ACC, MCC], Amygdala [Amyg], ventrolateral prefrontal cortex [vlPFC], dorsolateral prefrontal cortex [dlPFC], orbitofrontal cortex [OFC], primary and secondary somatosensory cortices [S1, S2], anterior and posterior insula [aI, pI]) for each age group (<32, 32-34, 34-36, 36-38, 38-40, 40-42, Adult). Connections for which 0% of babies passed the 'definite' connection threshold are marked as 'NC' (not connected).

		Age													
		<32		32-34		34-36		36-38		38-40		40-42		Adult	
Subnetwork	Connection	Mean	SE	Mean	SE	Mean	SE	Mean	SE	Mean	SE	Mean	SE	Mean	SE
Sensory	BG - SI	0.030	0.000	0.034	0.027	0.058	0.023	0.044	0.024	0.095	0.017	0.174	0.013	0.065	0.015
Sensory	BG - SII	-0.133	0.010	-0.034	0.044	0.024	0.023	0.052	0.025	0.127	0.018	0.122	0.013	0.076	0.017
Sensory	BG - pI	-0.031	0.045	0.003	0.058	0.063	0.027	0.132	0.028	0.208	0.018	0.218	0.014	0.065	0.017
Sensory	Thal - SI	-0.155	0.011	-0.016	0.035	0.051	0.022	0.041	0.028	0.144	0.018	0.168	0.012	0.049	0.015
Sensory	Thal - SII	-0.118	0.048	0.058	0.028	0.005	0.031	0.073	0.022	0.137	0.018	0.111	0.013	0.069	0.015
Sensory	Thal - pI	NC	NC	-0.111	0.031	-0.020	0.018	0.015	0.020	0.090	0.017	0.102	0.011	0.063	0.015
Sensory	SI - SII	NC	NC	-0.142	0.019	-0.045	0.017	0.017	0.014	0.082	0.014	0.102	0.010	-0.003	0.013
Sensory	SI - pI	NC	NC	-0.062	0.030	-0.031	0.019	0.019	0.023	0.067	0.016	0.024	0.011	0.049	0.016
Sensory	SII - pI	-0.213	0.000	-0.084	0.059	0.019	0.025	0.100	0.027	0.158	0.019	0.239	0.012	-0.005	0.010
Sensory & Affective	BG - Thal	-0.056	0.007	0.020	0.064	0.158	0.022	0.184	0.023	0.238	0.019	0.305	0.013	0.089	0.017
Affective	BG - Amyg	-0.172	0.014	-0.071	0.032	0.016	0.024	0.099	0.024	0.102	0.014	0.107	0.012	0.048	0.015
Affective	Thal - ACC	-0.043	0.033	0.157	0.000	-0.036	0.024	0.023	0.022	0.049	0.015	0.056	0.011	0.048	0.017
Affective	Thal - Amyg	-0.141	0.024	-0.052	0.036	-0.058	0.016	-0.003	0.019	0.001	0.014	0.019	0.011	0.043	0.015
Affective	Thal - aI	-0.130	0.014	-0.010	0.031	0.046	0.022	0.012	0.024	0.081	0.019	0.089	0.012	0.036	0.014
Affective	ACC - Amyg	-0.079	0.015	-0.064	0.047	-0.052	0.022	-0.050	0.019	0.003	0.015	0.038	0.011	0.040	0.014
Affective	ACC - aI	NC	NC	-0.159	0.000	-0.030	0.013	-0.045	0.020	0.016	0.015	-0.003	0.010	0.042	0.015
Affective	Amyg - aI	NC	NC	-0.089	0.049	-0.030	0.017	-0.034	0.022	0.034	0.015	0.054	0.010	0.058	0.014
Affective	BG - ACC	-0.133	0.008	-0.123	0.000	-0.105	0.016	-0.062	0.014	-0.018	0.012	-0.002	0.008	0.052	0.016
Affective & Cognitive	BG - aI	-0.205	0.000	-0.121	0.030	0.022	0.021	0.045	0.020	0.096	0.017	0.134	0.013	0.067	0.016
Cognitive	BG - MCC	-0.198	0.009	-0.133	0.023	0.024	0.024	0.021	0.023	0.079	0.019	0.106	0.012	0.038	0.015
Cognitive	BG - vlPFC	-0.189	0.006	-0.031	0.000	-0.132	0.014	-0.005	0.018	-0.018	0.015	0.017	0.010	0.037	0.016
Cognitive	BG - dlPFC	-0.013	0.028	0.034	0.037	-0.038	0.018	0.015	0.019	0.046	0.014	0.050	0.010	0.032	0.017
Cognitive	BG - OFC	0.001	0.039	-0.034	0.043	0.013	0.025	0.074	0.029	0.106	0.016	0.083	0.013	0.053	0.015
Cognitive	MCC - vlPFC	NC	NC	-0.158	0.000	-0.100	0.011	-0.048	0.017	-0.028	0.012	-0.042	0.008	0.051	0.014
Cognitive	MCC - dlPFC	NC	NC	-0.144	0.026	-0.072	0.016	-0.011	0.018	0.000	0.012	-0.006	0.008	0.015	0.012
Cognitive	MCC - OFC	-0.081	0.050	-0.035	0.037	0.000	0.023	0.064	0.021	0.085	0.015	0.068	0.011	0.052	0.016
Cognitive	MCC - aI	NC	NC	-0.183	0.001	-0.120	0.013	-0.061	0.019	-0.074	0.012	-0.034	0.009	0.049	0.015
Cognitive	vlPFC - dlPFC	NC	NC	NC	NC	NC	NC	-0.172	0.007	-0.138	0.005	-0.127	0.004	-0.003	0.008
Cognitive	vlPFC - OFC	NC	NC	NC	NC	-0.164	0.005	-0.100	0.016	-0.104	0.010	-0.059	0.007	0.029	0.010
Cognitive	vlPFC - aI	NC	NC	-0.129	0.000	-0.106	0.012	-0.074	0.016	-0.032	0.011	-0.034	0.007	0.003	0.012
Cognitive	dlPFC - OFC	NC	NC	NC	NC	-0.177	0.005	-0.167	0.007	-0.113	0.009	-0.113	0.005	0.008	0.012
Cognitive	dlPFC - aI	-0.194	0.011	-0.062	0.032	-0.065	0.019	-0.046	0.016	0.042	0.015	0.021	0.012	0.067	0.016
Cognitive	OFC - aI	0.028	0.003	-0.063	0.045	0.076	0.032	0.118	0.028	0.162	0.018	0.175	0.013	0.036	0.016
No subnetwrok	MCC - SI	0.099	0.000	0.066	0.061	0.233	0.027	0.242	0.030	0.243	0.017	0.261	0.014	0.053	0.014
No subnetwork	MCC - SII	-0.144	0.018	-0.150	0.044	-0.018	0.015	0.025	0.017	0.043	0.014	0.040	0.010	0.045	0.014
No subnetwork	MCC - pI	-0.155	0.000	NC	NC	-0.111	0.012	-0.048	0.017	-0.029	0.013	-0.031	0.008	0.055	0.015
No subnetwork	vlPFC - SI	-0.066	0.026	0.033	0.045	0.037	0.020	0.047	0.021	0.162	0.018	0.173	0.014	0.048	0.016
No subnetwork	vlPFC - SII	-0.131	0.012	-0.017	0.055	0.132	0.028	0.104	0.022	0.100	0.018	0.087	0.013	0.061	0.016
No subnetwork	vlPFC - pI	-0.145	0.020	-0.121	0.033	-0.071	0.018	-0.008	0.018	-0.003	0.011	-0.052	0.009	0.049	0.015
No subnetwork	dlPFC - SI	-0.103	0.051	0.027	0.050	0.038	0.026	0.071	0.031	0.129	0.017	0.151	0.013	0.078	0.017
No subnetwork	dlPFC - SII	-0.123	0.050	-0.147	0.028	-0.013	0.021	0.039	0.018	0.088	0.017	0.100	0.013	0.062	0.015
No subnetwork	dlPFC - pI	0.015	0.011	0.043	0.030	0.005	0.025	0.001	0.020	0.038	0.017	0.060	0.012	0.047	0.016
No subnetwork	OFC - SI	-0.066	0.031	0.023	0.021	-0.003	0.022	0.029	0.021	0.071	0.017	0.120	0.013	0.036	0.017
No subnetwork	OFC - SII	-0.125	0.030	-0.051	0.041	0.024	0.022	0.053	0.026	0.099	0.016	0.098	0.012	0.052	0.016
No subnetwork	OFC - pI	NC	NC	-0.147	0.023	-0.055	0.022	0.042	0.021	0.035	0.017	0.054	0.012	0.025	0.016
No subnetwork	Thal - MCC	-0.098	0.000	-0.037	0.007	-0.026	0.013	-0.045	0.016	-0.026	0.012	-0.016	0.009	0.042	0.014
No subnetwork	Thal - vlPFC	-0.192	0.000	-0.076	0.034	-0.058	0.020	0.014	0.019	0.073	0.016	0.090	0.012	0.062	0.016
No subnetwork	Thal - dlPFC	-0.151	0.008	-0.062	0.024	-0.060	0.019	0.007	0.022	0.042	0.014	0.058	0.011	0.048	0.014
No subnetwork	Thal - OFC	-0.004	0.038	0.014	0.041	0.041	0.022	0.047	0.028	0.128	0.016	0.129	0.013	0.053	0.017
No subnetwork	pI - aI	-0.203	0.000	-0.103	0.023	-0.096	0.016	-0.035	0.014	0.002	0.014	0.052	0.010	0.019	0.013
No subnetwork	SI - aI	-0.068	0.035	-0.006	0.035	0.056	0.025	0.096	0.033	0.123	0.021	0.150	0.013	0.077	0.015
No subnetwork	SII - aI	-0.179	0.000	-0.044	0.000	-0.084	0.016	-0.035	0.019	-0.027	0.012	0.038	0.011	0.034	0.013
No subnetwork	SI - ACC	NC	NC	-0.155	0.016	0.027	0.024	0.048	0.020	0.064	0.018	0.091	0.012	0.045	0.016
No subnetwork	SII - ACC	-0.064	0.021	0.005	0.045	0.010	0.020	0.058	0.021	0.077	0.015	0.104	0.013	0.052	0.017
No subnetwork	pI - ACC	0.010	0.059	-0.106	0.032	-0.037	0.024	0.031	0.020	0.045	0.017	0.069	0.012	0.049	0.015
No subnetwork	SI - Amyg	-0.099	0.013	-0.031	0.052	-0.009	0.023	0.000	0.019	0.056	0.016	0.065	0.012	0.077	0.015
No subnetwork	SII - Amyg	-0.085	0.064	-0.026	0.047	0.050	0.023	0.047	0.024	0.044	0.017	0.096	0.012	0.055	0.015
No subnetwork	pI - Amyg	-0.087	0.034	-0.069	0.018	0.048	0.027	-0.002	0.022	0.036	0.016	0.081	0.013	0.052	0.015
No subnetwork	Amyg - MCC	-0.186	0.002	NC	NC	-0.038	0.019	-0.011	0.022	0.027	0.012	-0.009	0.009	0.063	0.015
No subnetwork	Amyg - vlPFC	-0.086	0.015	-0.058	0.054	-0.019	0.019	-0.023	0.018	0.023	0.014	0.003	0.011	0.051	0.017
No subnetwork	Amyg - dlPFC	-0.120	0.027	-0.017	0.013	-0.065	0.017	-0.012	0.021	0.012	0.015	0.034	0.011	0.048	0.016
No subnetwork	Amyg - OFC	NC	NC	-0.149	0.019	-0.072	0.012	-0.064	0.018	0.003	0.013	-0.010	0.009	0.070	0.014
No subnetwork	ACC - MCC	NC	NC	-0.127	0.028	-0.090	0.013	-0.050	0.015	-0.051	0.010	-0.033	0.008	0.006	0.011
No subnetwork	ACC - vlPFC	NC	NC	-0.113	0.000	-0.052	0.016	-0.046	0.016	0.057	0.016	0.035	0.011	0.051	0.015
No subnetwork	ACC - dlPFC	NC	NC	NC	NC	-0.167	0.004	-0.120	0.010	-0.064	0.010	-0.089	0.007	0.005	0.013
No subnetwork	ACC - OFC	NC	NC	NC	NC	-0.127	0.009	-0.064	0.021	-0.052	0.013	-0.013	0.009	0.022	0.012

Supplementary Table 4. Pairwise comparisons of proportion of subjects with each connection and normalised strength of connection between subnetworks at different ages. Mean difference in proportion of subject with each connection (%) and normalised strength of connection (log_{10}(r-norm)) between subnetworks for each age group (Tukey corrected p-values for pairwise comparisons).

		Proportion of subjects with each connection (%)													
		<32 weeks		32 - 34 weeks		34 - 36 weeks		36 - 38 weeks		38 - 40 weeks		40 - 42 weeks		Adult	
		Mean Diff. (95% CI)	*p*	Mean Diff. (95% CI)	*p*	Mean Diff. (95% CI)	*p*	Mean Diff. (95% CI)	*p*	Mean Diff. (95% CI)	*p*	Mean Diff. (95% CI)	*p*	Mean Diff. (95% CI)	*p*
Network comparison	Sensory vs. Affective	-7.4 (-23.4 to 8.7)	.528	-0.2 (-16.3 to 15.8)	.999	9.3 (1.5 to 17.1)	.015	13.4 (6.2 to 20.5)	<.001	14.1 (9.5 to 18.6)	<.001	11.5 (8.1 to 14.8)	<.001	1.2 (-3.4 to 5.8)	.805
	Sensory vs. Cognitive	6.8 (-9.3 to 22.8)	.582	8.4 (-7.6 to 24.5)	.436	29.8 (22 to 37.6)	<.001	24.9 (17.7 to 32.1)	<.001	22.9 (18.4 to 27.4)	<.001	19.1 (15.8 to 22.5)	<.001	-4.6 (-9.1 to 0)	.052
	Affective vs. Cognitive	14.2 (-1.9 to 30.2)	.097	8.6 (-7.4 to 24.7)	.415	20.5 (12.7 to 28.3)	<.001	11.5 (4.4 to 18.7)	<.001	8.8 (4.3 to 13.4)	<.001	7.7 (4.3 to 11.1)	<.001	-5.8 (-10.4 to -1.2)	.009

		Strength of connections (log10 (r-norm))													
		<32 weeks		32 - 34 weeks		34 - 36 weeks		36 - 38 weeks		38 - 40 weeks		40 - 42 weeks		Adult	
		Mean Diff. (95% CI)	*p*	Mean Diff. (95% CI)	*p*	Mean Diff. (95% CI)	*p*	Mean Diff. (95% CI)	*p*	Mean Diff. (95% CI)	*p*	Mean Diff. (95% CI)	*p*	Mean Diff. (95% CI)	*p*
Network comparison	Sensory vs. Affective	-0.01 (-0.09 to 0.06)	.908	0.03 (-0.04 to 0.11)	.582	0.03 (-0.01 to 0.07)	.144	0.04 (0.01 to 0.08)	.005	0.07 (0.05 to 0.09)	<.001	0.07 (0.05 to 0.09)	<.001	0 (-0.03 to 0.02)	.889
	Sensory vs. Cognitive	-0.03 (-0.11 to 0.05)	.713	0.04 (-0.03 to 0.12)	.390	0.06 (0.02 to 0.10)	<.001	0.07 (0.03 to 0.10)	<.001	0.10 (0.08 to 0.12)	<.001	0.12 (0.11 to 0.14)	<.001	0.01 (-0.01 to 0.04)	.262
	Affective vs. Cognitive	-0.01 (-0.09 to 0.06)	.919	0.01 (-0.06 to 0.08)	.946	0.03 (0 to 0.07)	.104	0.02 (-0.01 to 0.06)	.239	0.03 (0.01 to 0.05)	<.001	0.05 (0.04 to 0.07)	<.001	0.02 (0 to 0.04)	.107

Supplementary Table 5. Pairwise comparisons of proportion of subjects with each connection and strength of connection between age groups within different subnetworks. Mean difference in proportion of subjects with each connection (%) and normalised strength of connection ($\log_{10}$(r-norm)) between age groups for each subnetwork (Tukey corrected p-values for pairwise comparisons).

	Proportion of subjects with each connection (%)						Strength of connections (log10 (r-norm))					
	Sensory		Affective		Cognitive		Sensory		Affective		Cognitive	
Age comparison (weeks PMA)	Mean Diff. (95% CI)	*p*	Mean Diff. (95% CI)	*p*	Mean Diff. (95% CI)	*p*	Mean Diff. (95% CI)	*p*	Mean Diff. (95% CI)	*p*	Mean Diff. (95% CI)	*p*
**<32 vs. 32-34**	-26.3 (-46.4 to -6.1)	.003	-19.1 (-39.3 to 1.1)	.077	-24.6 (-44.8 to -4.4)	.006	-0.01 (-0.2 to 0)	.043	-0.05 (-0.15 to 0.04)	.625	-0.03 (-0.13 to 0.07)	.968
**<32 vs. 34-36**	-46.8 (-62.7 to -30.9)	<.001	-30.2 (-46 to -14.3)	<.001	-23.8 (-39.7 to -8)	<.001	-0.16 (-0.23 to -0.08)	<.001	-0.11 (-0.19 to -0.04)	<.001	-0.07 (-0.15 to 0.01)	.117
**<32 vs. 36-38**	-53.4 (-69 to -37.7)	<.001	-32.6 (-48.3 to -17)	<.001	-35.3 (-50.9 to -19.6)	<.001	-0.19 (-0.27 to -0.11)	<.001	-0.14 (-0.21 to -0.06)	<.001	-0.10 (-0.18 to -0.02)	.002
**<32 vs. 38-40**	-58.1 (-72.9 to -43.2)	<.001	-36.6 (-51.5 to -21.8)	<.001	-41.9 (-56.8 to -27.1)	<.001	-0.26 (-0.33 to -0.19)	<.001	-0.17 (-0.24 to -0.11)	<.001	-0.13 (-0.20 to -0.06)	<.001
**<32 vs. 40-42**	-60.9 (-75.5 to -46.3)	<.001	-42 (-56.6 to -27.5)	<.001	-48.5 (-63.1 to -33.9)	<.001	-0.28 (-0.35 to -0.21)	<.001	-0.20 (-0.26 to -0.13)	<.001	-0.13 (-0.20 to -0.06)	<.001
**<32 vs. Adult**	-48.4 (-63.3 to -33.6)	<.001	-39.8 (-54.7 to -25)	<.001	-59.8 (-74.6 to -44.9)	<.001	-0.17 (-0.24 to -0.10)	<.001	-0.16 (-0.23 to -0.09)	<.001	-0.13 (-0.20 to -0.05)	<.001
**32-34 vs. 34-36**	-20.6 (-36.4 to -4.7)	.003	-11 (-26.9 to 4.8)	.381	0.82 (-15 to 16.7)	1	-0.06 (-0.13 to 0.02)	0.24	-0.06 (-0.13 to 0.01)	.195	-0.04 (-0.11 to 0.03)	.710
**32-34 vs. 36-38**	-27.1 (-42.7 to -11.5)	<.001	-13.5 (-29.1 to 2.1)	.143	-10.6 (-26.3 to 5)	.411	-0.10 (-0.17 to -0.02)	0.002	-0.08 (-0.16 to -0.01)	.014	-0.07 (-0.14 to 0)	.070
**32-34 vs. 38-40**	-31.8 (-46.6 to -17)	<.001	-17.5 (-32.4 to -2.7)	.009	-17.3 (-32.2 to -2.5)	.011	-0.16 (-0.23 to -0.09)	<.001	-0.12 (-0.19 to -0.05)	<.001	-0.10 (-0.17 to -0.03)	<.001
**32-34 vs. 40-42**	-34.6 (-49.2 to -20)	<.001	-22.9 (-37.5 to -8.3)	<.001	-23.9 (-38.5 to -9.3)	<.001	-0.18 (-0.25 to -0.11)	<.001	-0.14 (-0.21 to -0.07)	<.001	-0.10 (-0.17 to -0.03)	<.001
**32-34 vs. Adult**	-22.2 (-37 to -7.3)	<.001	-20.7 (-35.6 to -5.9)	<.001	-35.2 (-50 to -20.3)	<.001	-0.07 (-0.14 to 0)	0.043	-0.11 (-0.17 to -0.04)	<.001	-0.10 (-0.17 to -0.03)	<.001
**34-36 vs. 36-38**	-6.6 (-16 to 2.9)	.380	-2.5 (-11.9 to 7)	.987	-11.5 (-20.9 to -2)	.006	-0.04 (-0.08 to 0.01)	0.141	-0.02 (-0.07 to 0.02)	.736	-0.03 (-0.07 to 0.01)	.363
**34-36 vs. 38-40**	-11.3 (-19.3 to -3.2)	<.001	-6.5 (-14.5 to 1.5)	.205	-18.1 (-26.2 to -10.1)	<.001	-0.10 (-0.14 to -0.07)	<.001	-0.06 (-0.10 to -0.02)	<.001	-0.06 (-0.10 to -0.02)	<.001
**34-36 vs. 40-42**	-14.1 (-21.6 to -6.5)	<.001	-11.9 (-19.4 to -4.3)	<.001	-24.7 (-32.2 to -17.2)	<.001	-0.12 (-0.16 to -0.09)	<.001	-0.08 (-0.12 to -0.05)	<.001	-0.06 (-0.09 to -0.02)	<.001
**34-36 vs. Adult**	-1.6 (-9.67 to 6.4)	1	-9.7 (-17.7 to -1.7)	.007	-36 (-44 to -27.9)	<.001	-0.01 (-0.05 to 0.02)	0.955	-0.05 (-0.08 to -0.01)	.006	-0.06 (-0.10 to -0.02)	<.001
**36-38 vs. 38-40**	-4.7 (-12.3 to 2.9)	.523	-4 (-11.6 to 3.6)	.705	-6.7 (-14.2 to 0.9)	.123	-0.06 (-0.10 to -0.03)	<.001	-0.04 (-0.07 to 0)	.019	-0.03 (-0.06 to 0.01)	.182
**36-38 vs. 40-42**	-7.5 (-14.6 to -0.5)	.028	-9.4 (-16.5 to -2.4)	.002	-13.3 (-20.3 to -6.2)	<.001	-0.09 (-0.12 to -0.05)	<.001	-0.06 (-0.09 to -0.03)	<.001	-0.03 (-0.06 to 0)	.133
**36-38 vs. Adult**	4.9 (-2.7 to 12.5)	.469	-7.2 (-14.8 to 0.4)	.074	-24.5 (-32.1 to -16.9)	<.001	0.03 (-0.01 to 0.06)	0.343	-0.02 (-0.06 to 0.01)	.440	-0.03 (-0.06 to 0.01)	.223
**38-40 vs. 40-42**	-2.8 (-7.8 to 2.2)	.649	-5.4 (-10.4 to -0.4)	.026	-6.6 (-11.6 to -1.5)	.002	-0.02 (-0.04 to 0)	0.11	-0.02 (-0.04 to 0)	.107	0 (-0.02 to 0.02)	1
**38-40 vs. Adult**	9.6 (3.9 to 15.4)	<.001	-3.2 (-9 to 2.5)	.650	-17.8 (-23.6 to -12.1)	<.001	0.09 (0.06 to 0.12)	<.001	0.02 (-0.01 to 0.04)	.608	0 (-0.03 to 0.03)	1
**40-42 vs. Adult**	12.4 (7.3 to 17.5)	<.001	2.2 (-2.9 to 7.3)	.861	-11.3 (-16.3 to -6.2)	<.001	0.11 (0.09 to 0.13)	<.001	0.04 (0.01 to 0.06)	<.001	0 (-0.02 to 0.02)	1
